# Quercetin as a possible complementary therapy in multiple sclerosis: Anti-oxidative, anti-inflammatory and remyelination potential properties

**DOI:** 10.1016/j.heliyon.2023.e21741

**Published:** 2023-10-29

**Authors:** Parinaz Javanbakht, Farzane Rezaei Yazdi, Fatemeh Taghizadeh, Farnaz Khadivi, Hatef Ghasemi Hamidabadi, Iraj Ragerdi Kashani, Davood Zarini, Sina Mojaverrostami

**Affiliations:** a Department of Anatomy, School of Medicine, Tehran University of Medical Sciences, Tehran, Iran; b Department of Anatomy, School of Medicine, Shahrekord University of Medical Sciences, Shahrekord, Iran; c Department of Anatomy & Cell Biology, Faculty of Medicine, Mazandaran University of Medical Sciences, Sari, Iran

**Keywords:** Quercetin, Multiple sclerosis, Flavonoids, Remyelination, Anti-oxidants

## Abstract

Multiple sclerosis (MS) is a complex autoimmune disorder of the central nervous system (CNS) which causes various symptoms such as fatigue, dyscoordination weakness and visual weakness. The intricacy of the immune system and obscure etiology are the main reasons for the lack of a definite treatment for MS. Oxidative stress is one of the most important key factors in MS pathogenesis. It can enhance inflammation, neurodegeneration and autoimmune-mediated processes, which can lead to excessive demyelination and axonal disruption. Recently, promising effects of Quercetin as a non-pharmacological anti-oxidant therapy have been reported in preclinical studies of MS disease. In this review, we provide a compendium of preclinical and clinical studies that have investigated the effects of Quercetin on MS disease to evaluate its potential utility as a complementary therapy in MS. Quercetin treatment in MS disease not only protects the CNS against oxidative stress and neuroinflammation, but it also declines the demyelination process and promotes remyelination potential. The present study clarifies the reported knowledge on the beneficial effects of Quercetin against MS, with future implication as a neuroprotective complementary therapy.

## 1 Introduction

### 1.1 Multiple Sclerosis (MS)

Multiple Sclerosis (MS) was first defined in 1868 as a chronic demyelinating autoimmune disease of the central nervous system (CNS). However, MS can affect people at any age, but it is most commonly diagnosed in young women (between the ages of 20 and 40 years). The range of MS symptoms is diverse depending on the location of the demyelinating lesions in the CNS [1,2]. The etiology of MS is highly complex, and the combination of genetic and non-genetic factors likely leads to autoimmune disturbances and periodic attacks of the immune system in the CNS [3,4]. MS is not a fatal disease, but life expectancy is meaningfully reduced in MS patients compared to the healthy population [5]. In MS disease, inflammation can result in significant damage to the myelin sheaths surrounding the axons [6,7]. Also, oxidative stress-induced inflammatory responses can contribute to MS progression by damaging neurons and oligodendrocytes [8]. Four clinical courses have been identified for MS disease [9]. The relapsing-remitting MS (RRMS) usually affects about 85 % of MS patients. Primary progressive MS (PPMS) occurs after RRMS in some patients, and may occur concurrently with RRMS in others. Secondary progressive MS (SPMS) occurs when neurological functions worsen without any improvement and affects approximately 10 % of MS patients. Progressive relapsing MS (PRMS) affects fewer than 5 % of MS patients, and is characterized by a steady worsening of neurological functions [3,9].

The most important events in the pathogenesis of MS are demyelination, inflammation, reactive gliosis, and neuro-axonal injuries [10]. Autoreactive T cells that recognize self-antigens related to the myelin sheath pass through the blood-brain barrier (BBB) and secrete matrix metalloproteinases (MMPs), which can damage the extracellular matrix (ECM) [11]. The incursion of T cells into the CNS consequently activates the secondary inflammatory cells, such as macrophages [12]. The cellular analysis of MS lesions has shown that there are a large number of macrophages and CD^8+^T cells and a few number of CD4^+^T cells and B cells [13,14].

Many chemokine receptors (CCRs) are associated with either T helper 1 (Th1) or Th2 immune responses. Th1 cells (which promote inflammation) express CCR5, and Th2 cells (which promote anti-inflammation) express CCR3, CCR4, and CCR8 [[15], [16], [17]]. In MS disease, the migration of peripheral T cells is enhanced by the secretion of chemotactic chemokines such as RANTES (regulated on activation, normal T cell expressed and secreted) and MIP-1α (macrophage inflammatory protein). These chemokines are associated with the up-regulation of the CCR5 receptor [[18], [19], [20]]. Together, these data suggest that MS is likely to be associated with a variety of problems, such as microglial activation, blood-brain barrier destruction, immune responses, cytokine production, and oxidative stress [21].

The main pathological finding in MS is demyelination, which can be seen in the early stages of tissue destruction [14,22]. One of the special features of MS lesions is the survival and proliferation of oligodendrocyte progenitor cells (OPC). The number of these cells is greater than in normal conditions, but unfortunately, these cells cannot differentiate into mature myelin-producing cells [23]. In some lesions, surviving oligodendrocytes and those that have differentiated from OPCs may partially remyelinate damaged axons and create shadow plaques. As the lesions progress, astrocyte proliferation and activation become more apparent (Astrogliosis) [24]. Demyelinated axons are vulnerable to the attack of reactive oxygen species (ROS) and reactive nitrogen species (RNS) due to the loss of their external covering [25,26].

### 1.2 Oxidative stress in MS

Oxidative stress is a process that occurs due to the imbalance between antioxidant defenses and free radical-mediated injury. This imbalance can play an essential role in the progression of neurodegenerative diseases [27]. It has been suggested that in MS disease, inflammation can trigger the generation of oxygen and nitrogen free radical species, which can lead to oxidative stress and accelerate the progression of MS [28]. Activated microglia and astrocytes can produce ROS, NOS and inducible nitric oxide synthase (iNOS). Previously, it has been shown that neuroinflammation can induce oxidative stress in the CNS through two mechanisms: 1) the overproduction of ROS from glial cells, and 2) the arachidonic acid signaling pathway, which is activated by cyclooxygenase and lipoxygenase [29].

Oxidative stress can cause neural cell death by damaging DNA, lipids, and proteins [30]. Moreover, oxidative stress can lead to mitochondrial damage and functional disruption of the sodium-potassium ATPase pump, which reduces ATP production. This disruption can lead to potassium accumulation and subsequently apoptosis of the neurons [31]. NOS and ROS can damage the myelin sheath and activate macrophage/microglial cells. Additionally, the BBB function can be disorganized by the overproduction of NOS and ROS, which can worsen myelin degradation [32,33].

Oxidative stress can play a critical role in the pathogenesis of MS by BBB integrity and promoting the infiltration of leukocytes into the CNS. Eventually, the infiltrated leukocytes produce higher levels of ROS, which can lead to myelin destruction, oligodendrocyte damage, and axonal injury [28]. There is ample evidence that ROS overproduction is responsible for the progression and expansion of MS lesions [28]. The activation of the nuclear factor erythroid 2–related factor 2(Nrf2) signaling pathway can significantly prevent the pathogenesis of MS by controlling the levels of certain enzymes. In addition, it has been reported that the JNK and ERK signaling pathways can activate Nrf2 [34,35]. Nrf2 activates the Nrf2-ARE (erythroid-derived 2-like 2) antioxidant responsive element. This signaling pathway plays an essential role in protecting cells against oxidative stress, and it could be a promising target to activate antioxidant mechanisms in MS disease [36]. Previous studies have stated that paraoxonase 2 (PON2) has potent antioxidant activity, and its deficiency could lead to mitochondrial destruction [37].

There are two endogenous antioxidant defense systems: 1) Radical scavenger molecules (such as alpha tocopherol and glutathione) and 2) antioxidant enzymes (such as glutathione peroxidase, catalase, and superoxide dismutase) [29]. Among the different cells in the CNS, oligodendrocytes are very susceptible to oxidative stress due to their high metabolic activity, high iron content, and low antioxidant potential [8]. It has been reported that immature oligodendrocytes are more susceptible to oxidative stress than mature oligodendrocytes [38].

### 1.3 Quercetin

**Fig. 1:**
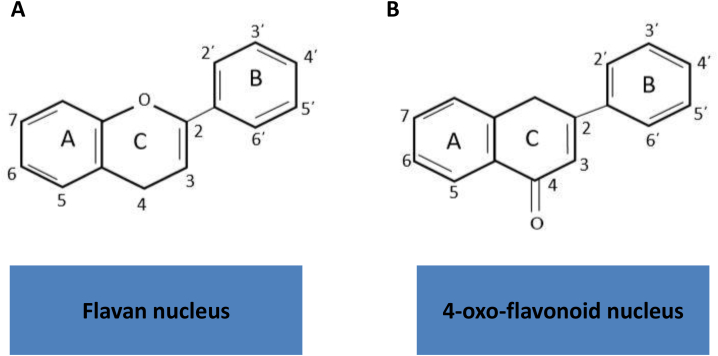
Basic flavonoid structures. (A) The Flavan nucleus structure and (B) 4-oxo-flavonoid nucleus.

**Fig. 2:**
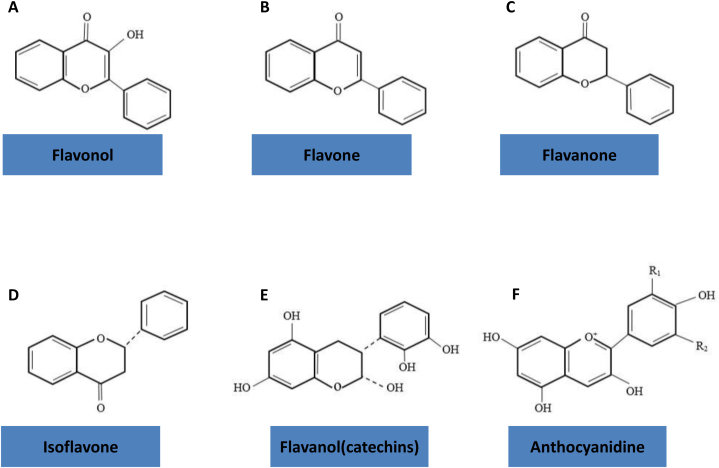
Structures of the major classes of flavonoid. (A) Flavonol, (B) Flavone,(C) Flavanone, (D) Isoflavone, (E) Flavanol and (F) Anthocyanidine.

Phytochemicals are a class of bioactive plant compounds that are present in various plants, such as vegetables and fruits [4,39]. Previous studies have shown that phytochemicals can reduce the risk of chronic diseases (such as cancer, hypertension, and diabetes) through various mechanisms, such as decreasing oxidative stress and inflammation [11]. Polyphenols are a subclass of phytochemicals with a large family of naturally occurring phenolic compounds [40]. Flavonoids are a class of polyphenolic secondary metabolites that are found in plants and are commonly consumed in the diets of humans [40]. Flavonoids are classified as a group of phytochemicals that have six subgroups (Flavonols, Flavanones, Flavones, Flavonols, Isoflavonoids, and Anthocyanidins) (Fig. 1, Fig. 2) [41]. The ability of flavonoids to cross the BBB, has attracted a great deal of attention for medicinal usage of these substances in the CNS [42].

The neuroprotective effects of flavonoids against various neurodegenerative disorders have been well-documented [41]. Flavonoids have a wide range of beneficial pharmaceutical properties, including anti-inflammatory, antioxidant, anti-carcinogenic, and anti-mutagenic effects [41]. Many studies have been conducted to evaluate the antioxidant effects of flavonoids on neurological diseases such as Alzheimer's and Parkinson's disease [42]. Recently, the study of the effects of polyphenols on MS disease has received much attention due to their immunomodulatory, anti-inflammatory, and antioxidant properties [43].

Flavonoids have three phenolic rings, which are named rings A, B, and C [44,45]. Flavonols) 3-hydroxy-2-phenylchromen-4-one (are characterized by a ketone group and a hydroxyl group in the C ring at position 3 [46]. Flavonols are found in vegetables (such as onions, kale, and tomatoes), fruits (such as apples and grapes), red wine, and tea [47]. Quercetin (C15H10O7) is an important subclass of Flavonols. Quercetin is abundant in the human daily diet such as fruits, vegetables and tea. Quercetin glycoside is formed when a glycosyl group (a sugar such as glucose, rhamnose, or rutinose) is attached to one of the OH groups of quercetin (commonly at position 3) [44,48,49]. The glycosyl group can alter the solubility, absorption, and biological activities of quercetin. Glycosylated forms of quercetin, such as rutin (quercetin-3-*O*-rutinoside), isoquercitrin (quercetin-3-*O*-glucoside), and hyperin (quercetin-3-*O*-galactoside), exist in different plant species [44,48,49].

**Fig. 3:**
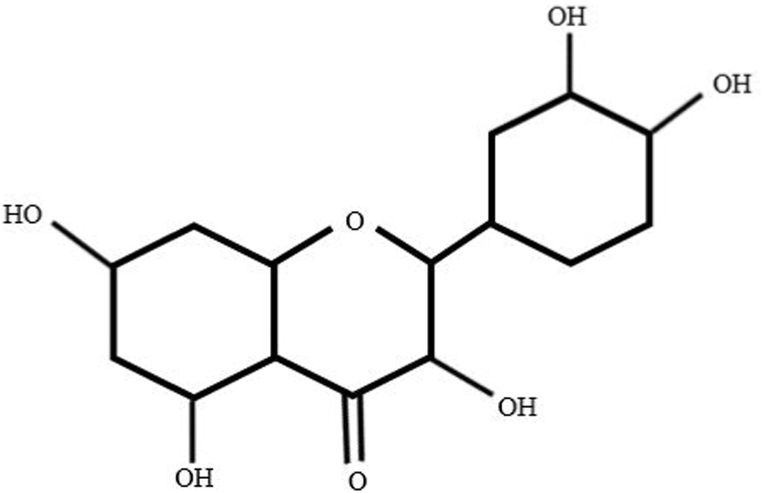
Molecular structure of Quercetin.

Quercetin is a member of the plant flavonoid family that can promote mental and physical functions through various pharmaceutical properties (Fig. 3). These properties include anti-cancer effects, prevention of platelet aggregation, reduction of serum fat levels, reduction of plasma insulin levels, and reduction of inflammatory and oxidative markers [50,51]. Recent evidence has demonstrated that quercetin has anti-inflammatory activity in addition to its antioxidant activity. This is not surprising, as quercetin has been used to treat many inflammatory-based disorders Until the present, many natural antioxidants have been used as a supplementary treatment to slow the progression of MS in preclinical and clinical studies [8]. Due to the promising effects of Quercetin in the CNS, recent studies have focused on its neuroprotective effects on various neurodegenerative disorders such as ischemia, traumatic damage, cognitive disability, Huntington's, Parkinson's, Alzheimer's and MS disease [[52], [53], [54]]. The antioxidant properties of Quercetin can inhibit lipid peroxidation and reduce the transcription of pro-inflammatory cytokines by scavenging free radicals [55]. It should be noted that, glycosylation itself can reduce the anti-inflammatory and antioxidative activities of the Quercetin [56]. The purpose of this review is to prepare a summary of the recent studies concerning the effects of Quercetin in MS disease along with a focus on the chemical and pharmacological properties of Quercetin and its possible neuroprotective effects.

## 2 Materials and methods

In the present study, we organized a narrative review based on preferred reporting items for systemic reviews and meta-analyses (PRISMA) guidelines. Scopus, Google scholar, PubMed and PubMed/Medline databases were searched, from inception to 1 June 2023, for studies that included the following keywords in the title and abstract (ti/ab): Multiple sclerosis [MeSH Terms], OR Cuprizone demyelination model [MeSH Terms] OR experimental autoimmune encephalomyelitis [MeSH Terms] OR demyelination model [MeSH Terms] AND Quercetin [MeSH Terms] OR Quercetin derivatives [MeSH Terms]. The reference lists of all selected articles were also checked to identify any additional relevant studies.

### 2.1 Neuroprotective efficacy of quercetin

**Fig. 4:**
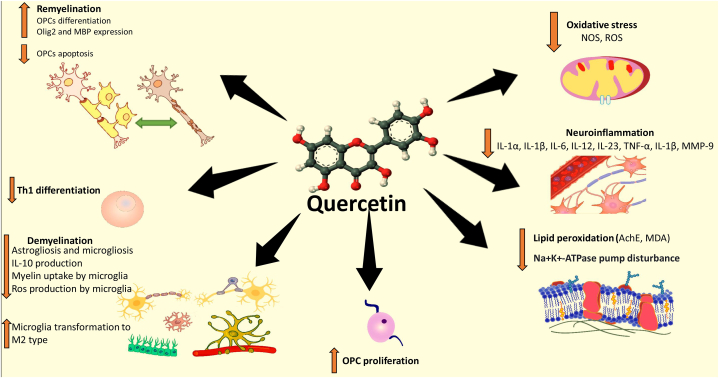
Neuroprotective effects of Quercetin in Multiple Sclerosis.

Recently, it has reported that berries are good source of Quercetin, with a high content of quercetin (6–158 mg/kg) (Fig. 4.) [57]. Quercetin can suppress oxidative stress and inflammation via modulating of nuclear factor erythroid 2–related factor 2 and heme oxygenase-1 signaling pathways Nrf2/HO1 and consequently suppress neurodegenerative disease progression [57]. Moreover, Quercetin is able to effectively prevents from aggregation of Alpha-synuclein (a-syn) through increase in the hydrophilicity of the covalently modified α-synucein [58]. Recent studies have showm that Quercetin significantly inhibited Okadaic acid induced tau pathology through suppressing hyperphosphorylation of tau protein and led to neuronal survival. Quercetin could inhibit tau hyperphosphorylation via mitogen-activated protein kinases (MAPKs) and phosphatidylinositol 3-kinase/Protein kinase B (PKB)/Glycogen synthase kinase-3 beta (PI3K/Akt/GSK3β) signaling pathways [59]. In addition, Quercetin can inhibit β-amyloid fibrillation and destabilize the preformed mature fibrils through reinforcing the hydrophobic interaction in the aromatic rings [60].

Previous studies showed that Quercetin had neuroprotective and anti-aging effects by activating Sirtuin 1(SIRT1) [61]. Previous reports have shown that Quercetin acts as a potent simulator of autophagy in Schwan cell [62].

Quercetin is able to attenuate behavioral and cognitive impairment in many neurodegenerative diseases [63]. Quercetin can ameliorate cognitive deficits through increasing learning and memory, reducing mitochondrial dysfunction, reducing senile plaques and increasing oxidative defense via activating AMP-activated protein kinase (AMPK) [64]. Quercetin also could reduce hypoxia induced memory dysfunction through its antioxidative and ani-apoptotic activities [65]. Quercetin significantly increased the number and length of neuritis via activation of P13K/AKT pathway [66]. Quercetin can increase the neuronal survival through enhancing the expression of neurotrophic proteins associated with neuritis outgrowth such as growth-associated protein43 (GAP-43), microtubule-associated protein (MAP) and tau, synaptophysin and Synapsin [67]. Also, Quercetin can exert neuroprotective function through JNK/activator protein-1(AP-1) signaling pathways, and this pathway can increase the expression of PON2 protein [68,69].

Quercetin has metal chelating activities and free radical scavenging properties. It can significantly prevent neuronal damage caused by oxidative stress. Hydroxyl substitutions, especially at the C3 (C-ring) and C5 (A ring) positions, lead to direct antioxidant activity [70]. Also, this compound can induce indirect antioxidant activity via inhibiting nitric oxide and xanthine oxidase synthase and cause the reduction of oxidative stress [36]. Previously, it has been well documented that Quercetin decreased oxidative stress-induced neuronal cell membrane damage more than vitamin C [71]. Altogether, the antioxidant feature of Quercetin can be considered as a biological benefit for protecting CNS during MS progression.

### 2.2 Potential implications of quercetin in MS

Oligodendrocytes demolition, myelin destruction and glial activation are attributed to the MS disease [72,73]. In patients with MS, endogenous remyelination often occurs. However, the differentiation and recruitment of OPCs are often not sufficient to overcome the demyelination condition [74]. It has been reported that quercetin can protect OPCs, reduce OPC apoptosis, and increase OPC proliferation and differentiation [75,76]. Application of this antioxidant considerably increases OPCs survival via PI3K/Akt signaling pathway [75]. When glial cells (Astrocytes and microglia) are activated, they release numerous inflammatory factors [77,78]. Quercetin can remarkably reduce the level of glial cells activation (reduce Astrogliosis and microgliosis) [79,80]. Several studies have shown that quercetin can decrease astrocyte activation by inhibiting the transition of the cell cycle from G1 to S phase, and prevent astrocyte proliferation by blocking ERK/focal adhesion kinase (FAK) [79,80].

Microglia are resident macrophages in the CNS which are divided into two phenotypes: M1 and M2 [81]. M1 microglia releases pro-inflammatory cytokines such as IL-1α, IL-1β, IL-6, IL-12, IL-23, TNF-α, and iNOS, while M2 microglia produce a series of neuroprotective and anti-inflammatory agents including IL-4 and IL-10 [[82], [83], [84]]. Quercetin has the capacity to promote microglial M2 phenotype conversion, and subsequently, M2 microglia secretes IL-4 which can reduce local inflammation and neuronal loss [84]. In addition, IL-10 secreted by M2 microglia can reduce the demyelination, promote remyelination, and lead to functional improvement [80,85].

Studies have shown that Quercetin treatment reduced the course of clinical paralysis by preventing the release of inflammatory cytokines and inhibiting the detrimental function of macrophages in the CNS [86]. Among the various mediators, IL-12 plays a pivotal role in the pathogenesis of MS [87,88]. IL-12 has an important role in blocking inflammation and preventing its symptoms. Quercetin can inhibit the transcription factor NF-κB, which leads to the decrease of MMP-9. The change in MMP-9/tissue inhibitor matrix metalloproteinase 1(TIMP-1) ratio in MS lesions is important because higher MMP-9 levels participate in BBB disruption and ECM degradation. Since MMP-9 can directly cause axonal damage, the effect of Quercetin on the reduction of the MMP-9/TIMP-1 ratio has attracted a lot of attention [89,90].

IL-12 activates Tyrosine kinase (TYK2), JAK2 and subsequently, STAT3 and STAT4 [91,92]. This signaling pathway is a key mechanism by which quercetin reduces inflammation via the JAK-STAT pathway. In the EAE model, it has been reported that quercetin inhibits the tyrosine phosphorylation of JAK kinases and STAT proteins, which interferes with Th1 cell differentiation [93]. Inhibition of the JAK-STAT pathway by quercetin resulted in a decrease in the proliferation of T cells and differentiation of Th1 cells [93].

### 2.3 Effects of quercetin in MS

#### 2.3.1 Animal studies on the potential benefits of quercetin in MS

Several studies have examined the efficacy of quercetin treatment on different animal models of MS (Table 1). In one study, Quercetin administration (50 mg/kg/day, oral gavage) was examined in an Ethidium bromide (0.1 %) induced demyelination rat model [94]. Quercetin was found to prevent additional demyelination, increase remyelination, improve locomotor activity in beam walking test, inhibit lipid peroxidation and prevent the inhibition of acetylcholinesterase (AChE) activity [94]. In the aforementioned study, the beneficial effects of Quercetin against demyelination model of MS were attributed to the close interaction of quercetin with the cholinergic neurotransmission [94]. Similarly, Carvalho et al. investigated the effect of Quercetin administration (50 mg/kg/day, oral gavage) in an Ethidium bromide (0.1 %) induced demyelination rat model [95]. They found that Quercetin could protect the function of Na+,K + -ATPase in the pons and cerebellum in both the demyelination and remyelination phases. They also reported that Quercetin could decrease oxidative stress and protect the function of AChE activity in whole blood and lymphocytes [95]. Another study was conducted to investigate the effects of Quercetin (25 and 50 mg/kg/day, oral gavage) on myelin repair in a lysolecithin (LPC) induced demyelination model (1 %, 2 μl) in the optic chiasm of rats [79]. The results showed that Quercetin treatment led to a significant decline in visual evoked potential (VEP) and demyelination area and augmented the remyelination process. The researchers indicated that quercetin can improve optic pathway function by reducing glial activation and inhibiting myelin sheath destruction [79].

**Table 1:** Quercetin effects on preclinical and clinical models of MS.

Author and year	Type of Study	Model of MS	Number of samples	Drug therapeutic dose and method of injection	Treatment period	Outcomes
Ahmadi et al., 2023	In-vitro	PBMC isolated from RRMS patients	8	100 μm in cell culture	48 h	Immunomodulatory effects of Quercetin Penta Acetate on Th17 cells of MS patients are more effective than Quercetin.
Tan et al., 2021	In vivo	Vascular dementia demyelination model in mice	40	60 mg/kg/day by intraperitoneal (i.p) injections	14 days	Quercetin facilitated microglia transformation into M2 phenotype, decreased production of proinflammatory factors (TNF-α and IL-1β) and enhanced microglial engulfment ability of myelin fragments.
Yu et al., 2020	In vivo	Cuprizone induced demyelination model in C57BL/6 J mice	50	50 and 100 mg/kg/day	35 days	Quercetin (50 and 100 mg/kg) could decrease demyelination in corpus callosum and increase remyelination via enhancing MBP and Olig2 expression.
Mirzazadeh et al., 2019	In vivo	EAE model in Wistar rat	30	10 mg/kg/day by intraperitoneal (i.p) injection	24 days	Quercetin decreased myeloperoxidase activity, nitric oxide and lipid peroxidation level in the serum.
Naeimi., 2019	In vivo	Lysolecithin induced demyelination model in optic chiasm of rat	_	25 and 50 mg/kg/day, oral gavage	7 and 14 days	Quercetin decreased the P1–N1 latency, increased the amplitude of VEPs waves and myelin repair was improved. Also, Quercetin moderated glial activation and reduced expression of GFAP and lba1 in optic chiasm.
Carvalho et al., 2018	In vivo	Ethidium bromide induced demyelination model in Wistar rat	80	50 mg/kg/day, oral gavage	7 and 21 days	Quercetin could protect the function of Na+,K + -ATPase in the pons and cerebellum in the both demyelination and remyelination phases. Also, Quercetin could protect the function of AChE activity in whole blood and lymphocytes in both demyelination and remyelination phases and regulate redox state.
Hashemian., 2018	In vivo	Lysolecithin demyelination model in optic chiasm of rat	_	25 mg/kg/day by intraperitoneal (i.p.) injections	7 and 14 days	Quercetin-loaded NPs reduced the extent of demyelination areas, attenuated glial activation and inflammation.
Naeimi et al., 2018	In vivo	Lysolecithin demyelination model in optic chiasm of rat	–	25 mg/kg, 50 mg/kg/day, by oral gavage	–	Quercetin reduced the delay of visual signals, alleviated the level of glial activation, decreased the extent of demyelination areas and increased the remyelination process and PLP expression.
Ghasemi-kasman., 2017	In vivo	Lysolecithin induced demyelination model in optic chiasm of rat	_	50 and 100 mg/kg/day, ip injection	7 and 14 days	Quercetin reduced the P1–N1 latency, increased the amplitude of VEPs waves, reduced the expression of GFAP, improved myelin repair. Also, Quercetin ameliorated astrocytes activation of optic chiasm.
Backman et al., 2014	In vivo	Ethidium bromide induced demyelination model in Wistar rat	80	50 mg/kg/day, oral gavage	7 and 21 days	Quercetin promoted locomotor recovery, decreased demyelination, increased remyelination, promoted AChE activity and inhibited lipidic peroxidation.
Liuzzi et al., 2011	In vitro	Cell culture of rat astrocyte activated with LPS Sera of RRMS patient	1 × 10^5^ cells/m 14	1–25 μg/ml in cell culture 50 μg/ml, in cell culture	overnight overnight	In LPS treated astrocytes the levels of MMP2 and MMP-9 were increased. Quercetin treatment of astrocytes could not inhibit the MMP-2 and MMP-9 levels in LPS activated astrocytes. Quercetin was able to signiﬁcantly inhibit the gelatinolytic activity up to 81 % in the serum of RRMS Patients.
Sternberg et al., 2008	In-vitro	PBMC isolated from RRMS patients	23	5–50 μM in cell culture	–	Quercetin, in a dose-dependent manner, reduced the proliferation of PBMC and regulated the level of IL-1β and TNF-α. Also, reduced the MMP-9/TIMP-1 ratio by reduce the MMP-9 production.
Muthian et al., 2004	In vivo	EAE model in SJL/J mice	–	50 and 100 μg/day by intraperitoneal (i.p) injection	25 days	Quercetin treatment indicated that in both in vivo and in vitro models could block IL-12 and led to the inhibition of T cell proliferation and Th1 differentiation.
Hendriks et al., 2003	In vitro	Myelin content of adult mice	–	300 μg in cell culture of macrophage and myelin	90 min	Quercetin could completely inhibit myelin uptake by macrophages and diminish ROS production.

In accordance with previous mentioned studies, some in-vitro studies have reported that Quercetin (3–27 μM) could decrease apoptosis, increase proliferation and differentiation of OPC after oxygen/glucose deprivation induced injury by the activating the PI3K/Akt signaling pathway [75,76]. Ghasemi-Kasman et al.**,** investigated the effects of Quercetin administration (50 and 100 mg/kg/day, ip injection) on astrocytes activation and remyelination in a LPC-induced (1 %, 2 μL) local demyelination in the optic chiasm of rats [96]. They reported that Quercetin increased the wave's amplitude of VEPs, decreased the P1–N1 delay, improved myelin repair, and reduced the expression of GFAP [96]. In a study by Hashemian et al. the anti-inflammatory effects of Quercetin-loaded nanoparticles (25 mg/kg/day, ip injection) were investigated in a LPC-induced (1 %, 2 μL) local demyelination in the optic chiasm of rats [97]. They indicated that quercetin-loaded nanoparticles can lead to a decrease in the extent of demyelination areas, reduced glial activation, and inflammation [97]. In another study, the effects of Quercetin on the activation of oligodendroglial linage, remyelination, visual and optic pathway and expression of genes related to the myelin formation were investigated in a LPC-induced focal demyelination model [98]. In the mentioned study, LPC (1 %, 2 μL) was injected into the rat optic chiasm for local demyelination, and treatment was performed with Quercetin (25 mg/kg, 50 mg/kg/day, by oral gavage). They reported that quercetin was able to increase the amplitude of VEPs waves and decrease the P1–N1 delay. They also reported that quercetin decreased lba1 and GFAP expressions, which are markers of microglia and astrocytes, respectively [98]. It has been previously reported that daily injection of 60 mg/kg Quercetin for 14 days (ip) in mice with vascular dementia facilitated microglia transformation into the M2 phenotype, reduced demyelination in the ventral hippocampus and mitigated neuropsychiatric deficits [80].

**Fig. 5:**
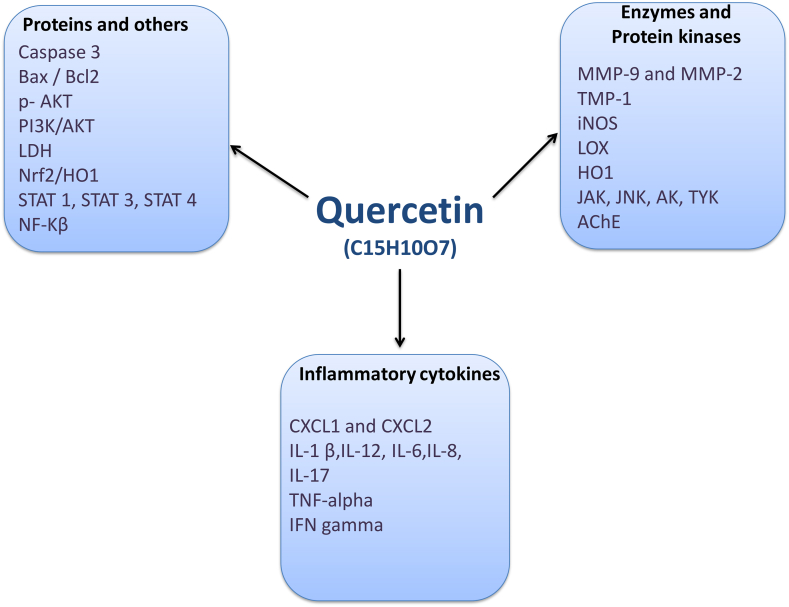
Multiple molecular targets of Quercetin against Multiple sclerosis.

In another study, Quercetin treatment (10 mg/kg/day, oral) in EAE model could decrease EAE progression via controlling myeloperoxidase activity, nitric oxide levels and lipid peroxidation in the sera [99]. Another study noted that daily administration of Quercetin (50 and 100 μg/day, ip) in SJL/J mice with EAE model ameliorated the disease progression via decreasing IL-12 production and neural Th1 differentiation [100]. They also found that in vitro treatment of activated T cells with quercetin decreased IL-12-induced T cell proliferation and Th1 differentiation (Fig. 5). Quercetin treatment (25, 50 and 100 mg/kg/day, oral) in Cuprizone induced demyelination model of C57 BL/6 mice could decrease demyelination in the corpus callosum [101]. They also reported that quercetin-treated mice, compared to non-treated mice, had higher MBP and Olig2 protein expressions in the corpus callosum, which were associated with the higher remyelination capacity [101]. In an in-vitro study, Quercetin effects were investigated in cultured condition of macrophages and mice myelin content. They found that Quercetin could completely inhibit myelin uptake by macrophages and diminish ROS production. Indeed, they have showed that Quercetin can protect CNS during MS progression via affecting macrophages functions [102].

#### 2.3.2 In vitro studies of the benefits of quercetin on PBMCs of MS patients

In a study by Sternberg et al. it was reported that quercetin, either alone or in combination with interferon-beta, can enhance immunomodulatory effects in PBMC obtained from MS patients [90] (Table 1). They assessed the PBMC proliferation and production of pro-inflammatory cytokines such as IL-1β, TNF-α, and the ratio of MMP-9 and its inhibitor, TIMP-1, in PBMC of MS patients following Quercetin treatment. The results of this study showed that quercetin decreased PBMC proliferation and the levels of IL-1β and TNF-α secretion. Quercetin also diminished the MMP-9/TIMP-1 ratio by decreasing MMP-9 production. When quercetin was used with interferon-beta, it had a better immunomodulatory effect and decreased TNF-α and MMP-9 levels [90]. Liuzzi et al. showed that Quercetin treatment could inhibit the activity of gelatinases involved in the course of the inﬂammatory responses observed in RRMS patients' sera [103]. However, they also reported that the levels of MMP2 and MMP-9 were significantly increased in the LPS-treated astrocytes culture of rats. Quercetin treatment of astrocytes did not inhibit the MMP-2 and MMP-9 levels in LPS-activated astrocytes [103]. Ahmadi et al. demonstrated that immunomodulatory effects of modified compound of Quercetin (Quercetin Penta Acetate) on Th17 cells proliferation and IL-17 gene expression in PBMC of MS patients are more effective than Quercetin [104]. They also recommended more clinical trials of the bioactive form of Quercetin as a supplementary treatment in MS patients.

## 3 Limitation

There is no specific clinical trial that has evaluated the effects of quercetin in MS patients. Most of the studies in this field have been conducted in animal models of MS or in vitro studies using PBMC from MS patients. The bioavailability of quercetin is very low. It can be improved by using several drug delivery methods, such as nanoparticles, inclusion complexes, liposomes, prodrugs, emulsions, phospholipid formulations, liposomes, nanocrystals, and micelles [105]. However, there are some concerns about using new drug delivery systems. These concerns include encapsulation efficiency, incomplete degradation of the carrier, lower drug loading, and accumulation in organs [106]. Although the beneficial effects of polyphenols such as Quercetin have been confirmed in pre-clinical studies as an adjuvant therapy for MS, they have not been considered in most clinical studies due to the lack of an understanding of the exact molecular mechanisms underlying these effects and the inconsistent findings. Certainly, more mechanistic and clinical studies are needed to shed light on the effects of quercetin in neurodegenerative diseases such as MS.

## 4 **Conclusion**

Quercetin can protect CNS by reducing oxidative stress and neuroinflammation. Quercetin has shown beneficial properties against MS progression in several preclinical studies. Recent studies have shown that resveratrol, curcumin, luteolin, quercetin, and hydroxytyrosol have demonstrated promising effects in preclinical studies. However, there is limited clinical evidence on the neuroprotective effects of some polyphenols, such as curcumin and epigallocatechin gallate, in MS patients [107]. Quercetin can reduce oxidative stress, inhibit the demyelination process, promote remyelination potential, improve optic pathway function, reduce glial activation, decrease apoptosis, enhance BBB integrity, and reduce inflammatory responses There is an urgent need for more clinical studies to determine the effects of quercetin in MS disease. These observations may lead to a better understanding of the neuroprotective role of quercetin and its importance as an adjuvant treatment for MS patients.

## Funding

This research did not receive any specific grant from funding agencies in the public, commercial, or not-for-profit sectors.

## Data availability statement

No data was used for the research described in the article.

## CRediT authorship contribution statement

**Parinaz Javanbakht:** Methodology, Validation, Writing – original draft. **Farzane Rezaei Yazdi:** Methodology, Software, Writing – review & editing. **Fatemeh Taghizadeh:** Data curation, Writing – review & editing. **Farnaz Khadivi:** Conceptualization, Writing – original draft, Writing – review & editing. **Hatef Ghasemi Hamidabadi:** Methodology, Validation. **Iraj Ragerdi Kashani:** Conceptualization. **Davood Zarini:** Writing – original draft, Writing – review & editing. **Sina Mojaverrostami:** Conceptualization, Supervision, Validation, Writing – original draft, Writing – review & editing.

## Declaration of competing interest

The authors declare that they have no known competing financial interests or personal relationships that could have appeared to influence the work reported in this paper.
